# Bisphenol A Promotes the Progression of Hormone-Sensitive Breast Cancers Through Several Inflammatory Pathways

**DOI:** 10.3390/cancers17142373

**Published:** 2025-07-17

**Authors:** Michael Thoene, Kamila Zglejc-Waszak, Marcin Jozwik, Joanna Wojtkiewicz

**Affiliations:** 1 Department of Medical Biology, School of Public Health, University of Warmia and Mazury in Olsztyn, 10-561 Olsztyn, Poland; 2 Department of Anatomy, Faculty of Medicine, Collegium Medicum, University of Warmia and Mazury in Olsztyn, 10-082 Olsztyn, Poland; kamila.zglejc@uwm.edu.pl; 3 Department of Gynecology and Obstetrics, Collegium Medicum, University of Warmia and Mazury in Olsztyn, 10-045 Olsztyn, Poland; marcin.jozwik@uwm.edu.pl; 4 Department of Human Physiology and Pathophysiology, School of Medicine, University of Warmia and Mazury in Olsztyn, 10-082 Olsztyn, Poland

**Keywords:** bisphenol A, breast cancer, inflammation, endocrine disrupting compounds, cancer stem cells, epithelial–mesenchymal transition, tumorigenesis, P53 mutation

## Abstract

**Background/Objectives**: Bisphenol A (BPA) is found throughout the environment and exposure to it has been shown to cause several health problems, including cancer. The problem with BPA is that it is a xenoestrogen that is chemically very similar to 17β-estradiol. Chronic exposure to BPA overstimulates the estrogen receptors and leads to inflammation that triggers several pathways leading to cancer progression. This is especially true in the case of hormone-sensitive breast cancers. This article reviewed the main pathways thought to be involved in the formation and/or progression of the most common forms of hormone-sensitive breast cancers due to BPA exposure. The main results were compiled and presented in tables along with a more detailed discussion of each pathway within the text. In most cases, chronic BPA exposure led to inflammation, which then triggered pathways leading to cancer stem cell formation and maintenance. In other cases, BPA exposure led to the formation of reactive oxygen species that damaged DNA and caused the formation of mutated p53 and tumorigenesis. **Conclusions**: The article summarizes the key pathways that are currently known, pertaining to how BPA leads to the progression and maintenance of breast cancer. The article then concludes by discussing how prenatal and perinatal BPA exposure may also predispose women to hormone-sensitive breast cancers later in life.

## Simple Summary

Bisphenol A is a monomer used to produce polymers used in the production of polycarbonate plastics and epoxy resins. It is found throughout the world and, under harsh conditions, tends to leak out of the polymer and enter into food and beverages. The monomers are structurally very similar to estrogen and tend to disrupt hormone signaling. In the case of breast cancer, these monomers overstimulate the estrogen receptors within the breast tissue, causing inflammation. This inflammation then triggers several pathways that lead to the formation and maintenance of cancer stem cells as well as causing tumorigenesis through DNA damage. This article has summarized the main pathways in a step-by-step manner within the text and has also presented those pathways in a table format for easy reference.

## 1. Introduction

**Figure 1:**
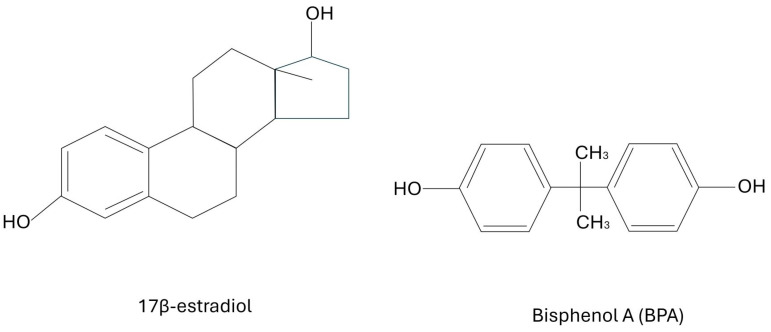
The structures of BPA and 17β-estradiol.

Bisphenol A (BPA) is an extremely common plastic that has recently been found to be an endocrine disrupting compound (EDC) that mimics estrogen. It has been used as an epoxy resin for several decades. Unfortunately, some of this polymer tends to leak its monomers into the surrounding environment. Its main use has been in commercially available products for consumers including food packaging, the lining inside of beverages and canned foods, baby formula packaging, baby bottles, and household food containers. It is also often used in dental prosthetics, and was commonly found in thermal paper such as sales receipts, although this has mainly been banned nowadays [1,2]. In December of 2024, the European Union banned the use of BPA in food contact materials. However, this particular EDC has been found in blood and urine samples of people across the entire globe; whether they live in rural developing regions or in highly advanced developed areas. The health effects range from abnormal childhood development to reproductive problems and cancers in adults [3]. Most typically, bisphenol binds to nuclear- and membrane-bound estrogen receptors (ERs), including G-protein coupled estrogen receptors (GPERs) that are often on the cell surface, as well as with estrogen receptors, known as ERα and ERβ, that are often found in the nucleus. BPA can bind to ERα and ERβ. Unfortunately, the exact mechanisms of which estrogen receptors are involved and exactly how they trigger certain pathways, such as inflammation, are not yet fully understood. Meanwhile, BPA could perhaps also interact with several other endocrine pathways. For example, there is a suspicion of BPA disrupting androgen receptors, thyroid hormone receptors, and other endocrine signaling pathways. There have been quite a few studies showing that this xenoestrogen may have significant effects at nanogram concentrations due to the disruption of normal hormonal signaling pathways [4]. In fact, studies have shown that BPA levels in urine vary from 0.36 to 2.07 μg/L and from 0.5 to 2.0 μg/L within blood samples [3,4]. Since BPA mimics estrogen, it has been theorized for at least a decade that it may be correlated with breast cancers. The key is the structural similarity between BPA and naturally occurring 17β-estradiol. Under natural conditions, the very potent form of estrogen (known as 17β-estradiol) is not always present, but is produced as needed for specific endocrine signaling. In the presence of chronic BPA exposure, the ERs, including GPERs, are continually activated since the receptors are binding BPA. Even when the estrogen receptors should be inactive, they are activated by the structural similarity of BPA to 17β-estradiol, which then causes disruption in endocrine signaling and the activation of subsequent inflammatory pathways. BPA shares a similar chemical backbone with 17β-estradiol, enabling it to mimic or interfere with natural estrogenic activity in the body. The structures of BPA and 17β-estradiol are shown below in Figure 1.

Describing cancers can be challenging due to a complex system of naming that may or may not include the type of tissue, the histology of the abnormal cells, the organ system of the neoplasm, the type of markers on the cell surfaces, and more. Even the same type of cancer may have more than one accepted name, which also adds to the complexity of cancer research. For this article, the focus will be on the most common forms of breast cancer, excluding atypical types, that are known to be hormone-sensitive. These cancers are adenocarcinomas, which originate from the epithelium that line the glandular tissue. These neoplasms may be invasive or in situ [5]. The most common origin of breast cancer is from the ducts that connect the lobules, and this accounts for approximately 80% of all breast cancers. This particular type is known as ductal carcinoma, and may be invasive ductal carcinoma (IDC) or ductal carcinoma in situ (DCIS). As the name suggests, the invasive form (IDC) can be life-threatening if not detected and treated very quickly [5]. DCIS is the contained form (in situ), which is less serious, but may still metastasize and become the more dangerous IDC. The second type of adenocarcinoma to be examined here will be invasive lobular carcinoma (ILC). ILC accounts for approximately 10% to 15% of all breast cancers and is the second most common form. ILC is almost always hormone-sensitive, while IDC and DCIS may or may not be hormone-sensitive [5,6]. The remaining five percent or so of breast cancers make up a lengthy list, but each particular one occurs less than one percent of the time; thereby making them less likely to have research about them with respect to BPA exposure.

IDC, ILC, and DCIS may or may not be hormone-sensitive, depending on the cell surface receptors that are present. These receptors may be located on the outer membrane surface, in the cytoplasm, and/or on the membrane of the nucleus. If these cancers express estrogen and/or progesterone receptors, it will make them responsive to hormonal therapies during treatment, but these receptors may have made certain cells susceptible to oncogenic mutation via EDCs such as BPA. There are three main classifications of breast cancer that depend on the type(s) of receptors present. The hormonal receptors can be ERs or progesterone receptors (PRs). Furthermore, there may be a receptor for human epithelial growth factor 2, which is not a hormone, but the receptor causes more of the protein to be produced that in turn causes oncogenic cells to grow at an accelerated rate. This receptor is known as HER2 [6,7]. Therefore, all breast cancers can be subdivided into: ER positive (ER+), ER negative (ER-), PR positive (PR+), PR negative (PR-), HER2 positive (HER2+), and HER2 negative (HER2-). Cells with none of these receptors are referred to as triple negative [7]. For this article, the emphasis will be placed upon the neoplasms that are ER+ and/or PR+, and the evidence currently found in the literature of how BPA is affecting them. If the HER2+ status is relevant, that will be mentioned as well.

This review article will discuss the mechanisms that have been separately published in various journals, and combine them into an overview of how BPA is promoting breast cancer through various biochemical mechanisms. These mechanisms often involve the overstimulation of hormone receptors within the breast tissue, thus leading to inflammation. That inflammation can then activate several biochemical pathways leading to decreased cellular adhesion and the formation of cancer stem cells (CSCs). Further pathways caused by inflammation can also alter the tumor microenvironment, allowing for the maintenance of those newly formed CSCs. Other inflammatory pathways cause oxidative stress and the formation of reactive oxygen species (ROS), which cause DNA damage, oncogene formation and epigenetic modifications that allow the breast cancer cells (BCCs) to evade the immune system. Also discussed in this article will be positive feedback loops caused by inflammation and ROS, as well as the role of mutated P53 caused by oxidative stress and DNA damage.

The articles cited in this review were selected by using the electronic database from the University of Warmia and Mazury. This database simultaneously searched across the majority of academic databases including, but not limited to, PubMed, Elsevier, Springer, ProQuest, and many more. The search phrases “bisphenol A” and “breast cancer” were initially used and returned 1632 articles, with 1258 of them being peer-reviewed. However, many of these articles were deemed to be more related to chemistry than to biology or medicine. Therefore, a new inquiry was performed using the terms “BPA” and “breast cancer”. There were 666 articles in general, with 596 of them peer-reviewed, and 582 of them were in the English language. After scanning titles and abstracts for mechanistic articles with a biochemical basis, 217 were initially selected for further analysis. Although many articles were very interesting, the focus was toward recurring major pathways that consistently arose. In total, 72 articles were chosen for more study and the most relevant were cited within the text. Prenatal and perinatal BPA exposure studies recurred so often that a section on that topic was also added into the article, since it seemed to be pertinent and of interest.

## 2. Mechanisms by Which Bisphenol Affects Hormones

As previously mentioned, BPA and other bisphenols can mimic estrogen, a key hormone in the regulation of various bodily functions, including reproductive health. They bind to estrogen receptors and can activate them, leading to effects similar to those of natural estrogen. This mimicry can disrupt normal hormonal signaling pathways and may promote the abnormal growth of hormone-sensitive tissues, such as those found in breast tissue. Additionally, bisphenols can interfere with the synthesis, secretion, and transport of other hormones, contributing to endocrine disruption. It has been suggested that these disruptions may increase the risk of hormone-sensitive cancers, such as ER+ and PR+ neoplasms [8].

Bisphenols, particularly BPA, are among the most studied endocrine disruptors due to their widespread use in plastics and their potential health impacts. They are known for their estrogenic activity, similar to substances like phthalates and certain pesticides, which can also mimic hormones. However, bisphenols may have a stronger affinity for estrogen receptors compared to some other chemicals, leading to more pronounced hormonal effects. Other endocrine disruptors, like heavy metals and flame retardants, may affect hormonal systems differently, either by altering hormone levels or by interfering with hormone signaling pathways. Overall, while bisphenols are particularly concerning due to their structural similarity to estrogen, other chemicals present varying degrees of risk to hormonal health as well [9].

## 3. Recent Scientific Studies Have Investigated the Relationship Between BPA Exposure and Hormone-Sensitive Breast Cancer

Some studies have indicated that BPA may contribute to the development of certain breast cancers, particularly hormone-sensitive ones [10]. There are a number of articles suggesting a direct causative association between BPA and IDC, DCIS, and/or ILC; however, most of these studies use BPA concentrations much higher than what is typically found in the environment. When the BPA concentrations are adjusted downward to more realistic levels found in the blood and urine of most patients, it seems that BPA predisposes epithelial cells to mutate and/or promotes these cancers to become more aggressive [11,12]. For example, BPA at typical in vivo levels has been shown to mutate DCIS from the non-invasive state to the more dangerous IDC form [12]. Other studies have also shown that prenatal and perinatal BPA exposure may predispose breast tissue to be more susceptible to carcinoma for women who use estrogen replacement therapy (ERT) during menopause [12,13].

**Figure 2:**
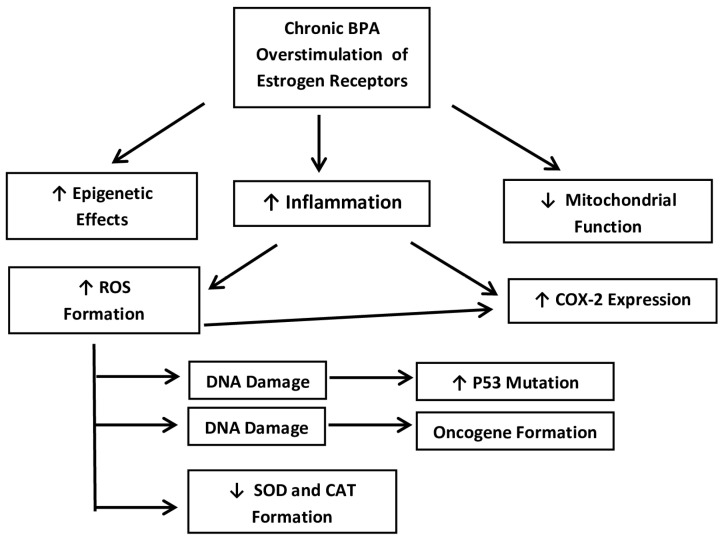
A simplified flow-chart of the main mechanistic pathways that are presented below. Source: own data.

Several mechanisms have been proposed at the molecular level to explain exactly how BPA may trigger the promotion of hormone-sensitive breast cancers. Essentially, it has been shown that chronic BPA exposure overstimulates the ER+ BCCs and activates several of the pathways that are discussed below [12,14]. At physiological concentrations, these may include the activation of CSCs via the cyclooxygenase-2 (COX2) pathway. Other pathways may transform DCIS into becoming invasive DC via decreased cell adhesion. Other theories include increasing levels of ROS, as well as decreased superoxide dismutase (SOD) and catalase (CAT). Other studies have also shown increased transcription activity of the p53 tumor suppressor, as well as the ZEB1 and WNT1 genes at the mRNA level. Most of these pathways begin with inflammation and the creation of ROS, which then activate various cascades leading to the progression of BCCs. These mechanisms seem to be especially relevant to ER+ ductal carcinoma (both IDC and DCIS), which are by far the most common forms [5]. Below, these pathways will be examined in more detail and the evidence for BPA predisposing and promoting these adenocarcinomas will be discussed. A flow chart has been provided in Figure 2 for clarification.

### 3.1. The Activation of Cancer Stem Cells via the COX-2 Pathway with Decreased Cell Adhesion

The COX-2 pathway plays a significant role in the maintenance and activation of CSCs in breast cancer. It contributes to tumor aggressiveness and even treatment resistance. Targeting this pathway is thought to be one future therapeutic strategy that may combat BCCs [15,16]. The COX-2 enzyme catalyzes the conversion of arachidonic acid to prostaglandins, and these lipid compounds have a role in inflammation and cell proliferation. In the context of breast cancer, the COX-2 pathway is involved in the activation of CSCs. This self-renewing subpopulation of tumor cells has the ability to differentiate into multiple cell types that comprise the tumor. CSCs are thought to be responsible for tumor initiation, progression, metastasis, and resistance to conventional therapies [16,17].

Clinically, high levels of COX-2 expression in breast cancer have been associated with poor prognosis, an increased risk of metastasis and reduced survival in general. This is why some research has focused on targeting the COX-2 pathway as a main strategy. The way the COX-2 pathway may affect neoplasms is by altering the tumor microenvironment. Increasing inflammation upregulates COX2 expression, which in turn increases prostaglandin production. In turn, this supports the maintenance and expansion of CSCs. The main prostaglandin formed is prostaglandin E2 (PGE2), and PGE2 has been shown to promote self-renewal and resistance to apoptosis in CSCs. PGE2 can also activate various signaling pathways, including the Wnt/β-catenin, PI3K/Akt, and STAT3 pathways, which are known to be involved in stem cell regulation [17,18]. As a consequence of the pathways listed above, COX-2 and its downstream effectors can induce epithelial–mesenchymal transition (EMT). EMT is a key step in the metastatic process where epithelial cells acquire increased motility and invasiveness, and thereby exhibit decreased cell adhesion, which leads to metastasis. This is most likely the cause of DCIS transforming into IDC. EMT is closely associated with the generation of CSCs by giving them mesenchymal characteristics [18]. The COX-2 pathway may also form and maintain a niche for the CSCs. This niche is a specialized microenvironment supporting CSC survival and function that protects the CSCs from immune surveillance.

BPA may upregulate the COX-2 pathway by several means. Firstly, BPA has been shown to induce an inflammatory response that can lead to the upregulation of COX-2 expression, increasing the production of prostaglandins, particularly PGE2 [14,16]. This in turn would also cause EMT, decreasing cell adhesion leading to an invasive form of breast cancer. Furthermore, the estrogenic activity of BPA could modulate the COX-2 pathway, since estrogen has been shown to do this [19]. Unfortunately, that particular mechanism is still not well understood. These are the main mechanisms that are currently believed to link BPA exposure to COX-2 upregulation and its role as a cancer-promoting agent. However, other factors may be at play as well, including oxidative stress that could lead to COX-2 upregulation [16,20]. Moreover, there could be epigenetic modifications that could also lead to COX-2 upregulation. BPA has been shown to methylate DNA and can modify histones as well [21,22,23]. These epigenetic changes could affect many things including inflammation and the expression of COX-2 [16]. Table 1 summarizes the above-mentioned pathways.

### 3.2. The Activation of Cancer Stem Cells via Reactive Oxygen Species and Epigenetic Changes

One of the main themes in the literature is that BPA initiates an inflammatory response as the xenoestrogen chronically overstimulates the ERs. The inflammatory response itself has been linked via multiple pathways to oxidative stress and the formation of ROS [20,24]. The inflammatory response itself may upregulate the COX-2 pathway as mentioned above, but the ROS may also directly damage DNA and lead to proto-oncogene mutations and subsequent oncogene formation; thus, creating BCCs [25]. The COX-2 pathway would then become involved as described above and lead to an invasive form of breast cancer via EMT.

Other research has shown that BPA can cause epigenetic effects. These post-transcriptional modifications mainly have been linked with DNA methylation and histone modifications [21,22,23]. During typical DNA proofreading after DNA replication, each cysteine residue in a CG dimer is methylated as evidence that the new strand of DNA has been duplicated accurately [22]. Therefore, these epigenetic modifications caused by BPA may hinder the proofreading ability of enzymes by making them appear that they have been accurately proofread and that no oncogenes are present. This would allow these oncogenes to persist through successive mitotic cycles without detection by the cellular machinery responsible for cell cycle checkpoints. This would help these BCCs to evade the immune system. Furthermore, the epigenetic modifications may also directly affect gene expression in ways not currently well-understood, in ways that may upregulate COX-2 expression or by activating other genes involved in inflammation. All of which may predispose the formation of BCCs. Table 2 summarizes the above-mentioned pathways.

### 3.3. The Activation of Cancer Stem Cells via Decreased SOD and CAT

The main function of the enzymes known as SOD and CAT are to break down harmful molecules that may generate ROS. As can be seen from the section above, ROS generation causes multiple negative effects at the cellular level. A clearer definition of what SOD and CAT are, plus their exact functions, are presented below.

BPA can decrease SOD and CAT activity through multiple mechanisms. This can include ROS generation that overwhelms the system, transcriptional repression, mitochondrial dysfunction, and/or direct enzyme inhibition [7,14,26]. One way that BPA can increase ROS production dramatically is by impairing mitochondrial function. This leads to increased electron leakage from the electron transport chain (ETC) and elevates ROS production. Mitochondrial dysfunction can reduce the synthesis of SOD2 (the mitochondrial isoform of SOD) and other antioxidants. These effects contribute to oxidative stress and may promote cancer development and progression. Superoxide dismutase (SOD) and catalase (CAT) are both enzymes involved in reducing the amount of ROS within the system. SOD produces less reactive hydrogen peroxide (H_2_O_2_) and oxygen (O_2_) from very damaging superoxide radicals (O_2_^−^), while CAT then further breaks down H_2_O_2_ into water (H_2_O) and oxygen (O_2_). This is crucial in preventing too many ROS to accumulate [27]. Therefore, decreased SOD and CAT activity will increase the amount of ROS, thus leading to oxidative stress and the inflammation that may trigger BCCs, as referenced above. Furthermore, the opposite may also be true. Inflammation can also increase ROS as well, and since BPA has been shown to cause inflammation, this could be one of the pathways of how BPA influences BCC formation. BPA triggers inflammation, the SOD and CAT enzymes are downregulated, ROS levels rise, and the following pathways come into play.

The mechanisms linking CSC activation and decreased SOD and CAT are varied. The elevated ROS, due to decreased SOD and CAT, can activate signaling pathways that include nuclear factor kappa B (NF-κB), hypoxia-inducible factor 1-alpha (HIF-1α), and the Wnt/β-catenin pathway. These have been shown to promote self-renewal, survival, and resistance to therapy, which are characteristics of CSCs [20]. NF-κB is an, evolutionarily, very old enzyme involved in the innate immune system. Its main function is to organize a resistance at the cellular level against pathogens [28]. NF-κB acts as a master regulator, due to its evolutionary age, and is involved with inflammation [29] that may then become involved with the COX2 Pathway with decreased cell adhesion, as described above. HIF-1α is an enzyme involved in oxygen regulation. If cells become hypoxic, then HIF-1α is expressed and can deliver needed oxygen through various mechanisms [30]. However, it is a very versatile enzyme and also plays a role in cell survival and proliferation, which favors CSC maintenance. It has been shown that ROS can stabilize HIF-1α under normal oxygen levels, thus activating it without hypoxia. This favors the maintenance of CSCs [30]. Meanwhile, the Wnt/β-catenin pathway is normally important for homeostasis and embryonic development. However, if this pathway becomes deregulated, it has been shown to cause tumor development [31]. Most of the dysregulations of this pathway seem to come from mutations in one or more of the many enzymes that make up the cascade. Once the pathway is deregulated, it often leads to malignant transformations, tumorigenesis, metastasis, a more rapid spread, and drug resistance. This is thought to be caused by the deregulated pathway disrupting the immune system; especially the immune checkpoint blockers [31]. Exactly how these mutations may appear is uncertain, but increased ROS has been shown to cause DNA damage.

BPA may be causing the increased inflammation, lowering SOD and CAT levels, and triggering the higher ROS levels. The ROS levels in turn activate the pathways described above that promote everything from tumorigenesis to metastasis. Therefore, the one link that all of these pathways have are increased ROS, caused initially by BPA-induced inflammation. However, BPA is not the only trigger for inflammation in our environment, but BPA may be helping other pro-inflammatory factors to enhance or promote BCCs. Table 3 summarizes the above-mentioned pathways.

### 3.4. Increased ZEB1 and WNT1 Aided by the Loss or Mutation of p53 Promote Increased CSCs

In this section, the main idea is that the very important tumor suppressor known as p53 becomes mutated and may even help the progression of cancer, rather than blocking it. ROS, as described above, damages DNA and can lead to a mutated p53. The mutated p53 then becomes involved in several pathways, but one of the key transformational pathways involves the enzyme known as ZEB1. ZEB1 is important for embryonic development, but in adult cells, it causes epithelial cells to lose adhesion and become cancerous. Normal p53 suppresses ZEB1, but mutated forms have been shown to actually increase the expression of ZEB1. This is explained in detail below.

There is a link between CSCs and mutated or missing p53. p53 is the most important tumor suppressor in all carcinomas, including breast cancer, and works to suppress mitosis if there is an error in DNA repair [32]. This stops any rogue cell from becoming a tumor. However, more than fifty percent of all cancer cells, including BCCs, have mutations in their p53 that not only allow mutated cells to form, but often actively help to promote tumorigenesis and metastasis [32]. Mutated p53 also inhibits differentiation, and this helps maintain CSC populations. Meanwhile, ZEB1 is a transcription factor involved in EMT, as described previously. The loss of cellular adhesion, especially by repressing E-cadherin, aids the development of CSCs. Furthermore, WNT1 is an important ligand in the WNT/β-catenin pathway, mentioned previously, which is crucial for the maintenance of CSCs. When WNT1 activates β-catenin, it promotes the expression of genes such as Cyclin D1 and c-Myc (implicated in stem cell reprogramming) that promote the self-renewal and even chemoresistance typically found in CSCs [33].

Therefore, while properly functioning p53 will suppress ZEB1 formation, the mutated p53 will actually enhance ZEB1 expression. The increased ZEB1 then leads to decreased cell adhesion via EMT, thus promoting newly formed CSCs. Furthermore, the more ZEB1 that is available, the more WNT signaling will be enhanced, and all of this helps to drive CSC development and expansion. In an added feedback loop, the WNT that becomes activated (via the β-catenin mechanism above) will repress any properly functioning p53 [32]. How p53 becomes mutated in any carcinoma is usually cited as being from a DNA mutation, either by a virus, chemical exposure, radiation, or random chance. In the case of BCCs and BPA exposure, the exact cause of the p53 mutation has not been well-documented [34,35]. However, DNA mutation of the p53 gene from ROS caused by BPA-induced inflammation cannot be ruled out. These pathways not only promote CSC formation, but are also involved in therapy resistance. Therefore, drugs that may restore wild-type p53 and block ZEB1 and WNT could help reduce CSCs, thereby increasing the effectiveness of therapies. Table 4 summarizes the above-mentioned pathways.

### 3.5. Prenatal and Perinatal BPA Exposure May Predispose Women to Later Breast Cancer

Quite a bit of clinical research has concluded that early-life BPA exposure may predispose women to contract breast cancer later in life. There are only a few mechanistic theories of how this happens, but clinically it has been recorded in the literature. It has been shown that BPA can pass through the placental barrier during pregnancy and affect fetal development [12,13]. A study by Paulose et al. showed in mice that it is possible for women who were exposed to BPA in early life to be significantly more likely to develop breast cancer during menopause if they used ERT [36]. While some studies have shown, generally, that BPA definitely impacts fetal development, it has been more challenging to explain the exact mechanisms [12,13]. The best explanation to date is that the developing fetus only contains estrogen receptors in what will later become breast tissue. Therefore, the fetal BPA exposure triggers changes in the COX-2 pathway and sensitizes those ER+ tissues to be more susceptible to xenoestrogens or even to ERT in the future [12,26,36]. Since fetal exposure to BPA seems to only predispose ER+ cells to more likely become BCCs, without immediately transforming them in young women, it is unlikely that BPA is the sole cause of these transformations of healthy cells into CSCs and BCCs. It is more likely that BPA exposure is a major factor at physiological levels in vivo. This is not to downplay the seriousness of BPA in our environment. BPA exposure is definitely a major risk factor in the formation of most breast cancers, but it may be working in conjunction with something else in our environment as well, and that should also be further investigated.

## 4. Discussion

### 4.1. How BPA Triggers Inflammation That May Predispose Hormone-Sensitive Breast Cancer

As previously stated, the endocrine-disrupting chemical BPA so closely resembles estrogen (specifically it most closely resembles 17β-estradiol) that it promotes inflammation in hormone-sensitive BCCs by overstimulating the estrogen receptors [37]. Through multiple molecular mechanisms, this can lead to the formation of CSCs and BCCs. As a nearly chemically identical match to 17β-estradiol, the xenoestrogen BPA continually triggers the ER+ hormone receptors (commonly referred to as ERα/ERβ) in breast tissue [5,6]. This overstimulation leads to the activation of signaling pathways that are ER-dependent, and this promotes or predisposes BCC proliferation and survival. Moreover, this chronic ER activation causes the pro-inflammatory gene expression that has been described above, especially the COX-2 pathway with subsequent ROS formation leading to the rise in CSCs. The chronic over-activation of these estrogen receptors also triggers pro-inflammatory cytokines, especially the key inflammatory transcription factors NF-κB, HIF-1α and the Wnt/β-catenin pathway that were mentioned previously [28,29,30]. There are also other cytokines involved in the pro-inflammatory microenvironment that make it possible to sustain BCCs. These include, but are not limited to, TNF-α, IL-6, and IL-8. These are mainly thought to promote JAK/STAT signaling, which has been shown to sustain inflammation that helps in cell proliferation [38,39,40]. It is possible that the overstimulation of ERs by BPA is helping to promote these cytokines, but more research into those pathways with regard to BPA needs to be conducted.

Aside from the xenoestrogen overstimulation of ERs triggering inflammatory pathways, this overstimulation can also create ROS that promote the formation of CSCs and BCCs as well. By definition, ROS causes oxidative stress. Not only will this activate most of the pathways mentioned above, but it can also directly cause DNA damage and mutations, such as creating a mutated form of p53. As discussed previously, mutated p53 is found in more than half of all carcinomas, including breast cancer, and will actively promote more aggressive BCCs [32,34]. Finally, BPA has been shown to alter DNA methylation, triggering epigenetic modifications. These epigenetic changes have been shown to both silence tumor suppressors such as BRCA1 and p53, as well as activate oncogenes such as Cyclin D1 and c-Myc, as mentioned previously [33,35,36].

### 4.2. A Discussion of the Specific Pathways Involved

The COX-2 pathway is perhaps the major pathway involved in BPA-induced breast cancer. So much so that, clinically, COX-2 expression has been a target for breast cancer therapy [15,16]. In fact, increased COX-2 expression has been linked with a poorer-than-average prognosis. As the BPA overstimulates receptors and causes inflammation, the COX-2 pathway becomes overly activated, which then leads to increased PGE2 expression. The overabundance of PGE2 then activates several other pathways that are significant in activating and maintaining CSCs. The currently known pathways that increased PGE2 triggers include the Wnt/β-catenin, PI3K/Akt, and STAT3 pathways. These three mechanisms are known to activate and maintain CSCs, as referenced above. However, lesser-known pathways may also be triggered, and that is worthy of future research. Besides the activation of these pathways, upregulated COX-2 expression and the subsequent PGE2 overexpression has been shown to increase EMT, thus making BCCs more mobile and aggressive. Less understood, but still possible, are mechanisms where increased ROS caused by chronic BPA exposure could itself directly increase COX-2 expression, bypassing the inflammation step, or epigenetic changes induced by chronic BPA exposure could do the same. More research into these mechanisms needs to be conducted.

ROS formation is also a major mechanism as well. As previously mentioned, the increased ROS formation can increase the expression of COX-2; however, ROS formation caused by BPA-induced inflammation can directly damage DNA leading to the mutation of proto-oncogenes. Epigenetic changes are also a factor. Even without ROS formation, BPA has been shown to directly cause epigenetic effects by methylating DNA that was not properly proofread and/or by modifying histones. This may allow BCCs to evade the immune system [22]. Although it has been shown that BPA causes these epigenetic effects, more research needs to be performed in order to more clearly define the biochemical mechanisms underlying those effects.

BPA can also decrease the expression of SOD2 and CAT enzymes as well, which causes increased ROS formation. This mainly occurs by BPA directly interfering with the mitochondria, which then causes electron leakage. Those leaked electrons then create an increased number of ROS. The increased ROS levels then overwhelm the system and lead to a decreased expression of SOD2 and CAT enzymes, which in turn increases the level of ROS even further. The main functions of SOD2 and CAT are to break down ROS. The electron leakage from the mitochondria overwhelms the system. Furthermore, BPA-induced inflammation can directly create ROS, which then suppress SOD2 and CAT expression, which may then lead to several other alternative pathways, often causing another positive feedback loop. Finally, the same ROS described above may activate the NF-κB, HIF-1α, and Wnt/β-catenin pathways that are known to increase CSC formation.

Furthermore, increased ROS formation caused by chronic BPA exposure may directly damage DNA. Not only may proto-oncogenes be mutated into oncogenes, but ROS may mutate the p53 enzyme that is very much necessary for control of the cell cycle. Mutated p53 may increase tumorigenesis and lead to CSCs. In fact, it has been shown that mutated p53 may often stimulate the growth of BCCs. On top of this, mutated p53 has been shown to stimulate various pathways that lead to increased Cyclin D1 and c-Myc that are responsible for the self-renewal of CSCs.

### 4.3. The Long Lasting Effects and Future Implications of BPA Exposure

BPA also tends to have lasting effects. Research has shown that prenatal and perinatal BPA exposure may lead to a greater sensitivity later in life to developing breast cancer. These pathways and mechanisms have not yet been clearly defined and we are not sure if BPA is acting alone or in conjunction with other environmental factors. This is definitely an area for future research. In our research on this topic, there were a few other proposed mechanisms and pathways, but the ones covered above were the best documented and seemed to be the most probable.

While most of the biochemical mechanisms described above were not performed at a clinical level, they are applicable due to their working at a cellular and/or enzymatic level. The pathways are known to be relevant to human cancer progression. However, in the case of prenatal and perinatal BPA exposure studies, most were performed at the clinical level with the exception of the studies linking very early BPA exposure to cancer development during menopause. Those studies were performed in rodents, for the purpose of time constraints.

It is also worth mentioning that there are several BPA replacements used in “BPA-free” products. These replacement bisphenols have similar structures to BPA, and it is unclear if these alternatives are actually safer than BPA or if society is simply switching one EDC for another type of EDC. This is a topic for future study, since the investigations into BPA alternatives is still a very new topic.

## 5. Conclusions

This review article has shown the main ways in which BPA may promote and worsen the formation of the most common hormone-sensitive breast cancers. The overall mechanism is an overstimulation of hormone-sensitive receptors that cause inflammation and subsequent ROS. The ROS may then become involved in positive feedback loops, which continually worsen the condition by creating more inflammation, may activate various biochemical pathways (such as the COX-2 and Wnt/β-catenin pathways) or it may directly damage DNA, in turn creating oncogenes and/or mutating the p53 checkpoint inhibitor enzyme. It is also quite likely that many or all of these mechanisms may be happening at the same time. That is an area where a more holistic view of breast cancer and EDCs could be developed.

Most of the literature focuses on the ER (ERα/ERβ) with little or no discussion of cells with active PR. Most of the available research focuses on estrogen receptors and even certain specific types of estrogen receptors without giving much regard to the PR+ or even HER2+ cancer subtypes. At the molecular level, the most likely culprit would be that the overstimulation of estrogen receptors causes imbalances, which in turn leads to ROS formation. However, it is possible that BPA is also binding PR in a similar way, and causing these same pathways. More research needs to be conducted, and a good start would be to see if these same pathways are active in PR+ only cancer cells lines. Perhaps using PR+ cell lines with removed estrogen receptors would answer this question. For example, if the PR+ only cell lines reacted exactly the same as ER+, then the same pathways are being activated. If the PR+ cell lines behaved differently, then other pathways related to hormone imbalance would be more likely. Likewise, there was little or no discussion of cells with human epithelial growth factor 2 (HER2+). More research should be performed with regard to HER2+ cells in order to see if they are also subject to some of these pathways. This is more challenging, since HER2 is a protein receptor known more for quick cell growth. However, a similar experiment with HER2+ cell lines, as proposed for PR+ cell lines, could be performed.

## Figures and Tables

**Table 1:** Summary of the effects of the COX-2 pathway in promoting breast cancer.

Reference	BPA Overstimulates Estrogen Receptors Causing the Release of Inflammatory Cytokines, Causing:
Pang et al., 2016 [17]	**↑** Inflammation **→ ↑** COX-2 Expression **→ ↑** PGE2 formation **→ ↑** CSC proliferation
Wu et al., 2017 [18]	**↑** Inflammation **→ ↑** COX-2 Expression **→ ↑** PGE2 formation **→ ↑** alternative pathways **→** CSC maintenance
Harris et al., 2014 [16]	**↑** Inflammation **→ ↑** COX-2 Expression **→ ↑** PGE2 formation **→ ↑** EMT **→ ↓** cell adhesion **→** **↑** Breast cancer invasiveness

**Table 2:** Summary of the effects of ROS and epigenetic changes affecting formation of CSCs.

Reference	BPA Overstimulates Estrogen Receptors Causing:
Biswas, 2016 [24]	**↑** Inflammation **→ ↑** Oxidative stress **→ ↑** ROS formation **→ ↑** COX-2 pathway
Srinivas et al., 2019 [25]	**↑** Inflammation **→ ↑** Oxidative stress **→ ↑** ROS formation **→ ↑** DNA Damage **→ ↑** Oncogene formation
Qin et al., 2021 [22]	**↑** Epigenetic Effects **→ ↑** Immune System Evasion

**Table 3:** Summary of the effects of decreased SOD and CAT and the formation of cancer stem cells after BPA.

Reference	BPA Overstimulates Estrogen Receptors Causing:
Zhu et al., 2025 [27]	**↓** Mitochondrial Function → ↑ Electron Leakage → ↑ ROS formation → **↓** SOD2 and CAT Formation
Liu et al., 2022 [31]	**↑** Inflammation **→ ↑** ROS formation **→ ↓** SOD2 and CAT Formation **→ ↑** alternative pathways = Positive Feedback loop
Albensi, 2019 [28]	**↑** Inflammation **→ ↑** ROS formation **→ ↓** SOD2 and CAT Formation **→ ↑** NF-κB, HIF-1α and Wnt/β-catenin pathways **→ ↑** CSC Formation

**Table 4:** Summary of the effects of mutated p53 and the formation of cancer stem cells after BPA.

Reference	BPA Overstimulates Estrogen Receptors Causing the Formation of ROS, Which Causes:
Rivlin et al., 2011 [32]	↑ DNA Damage **→** ↑ p53 Mutation → ↑ Tumorigenesis
Rivlin et al., 2011 [32]	↑ DNA Damage **→** ↑ p53 Mutation → **↓** Differentiation **→** ↑ CSC Populations
Arizti et al., 2000 [35]	↑ DNA Damage **→** ↑ p53 Mutation → **↑** Zeb1 Formation **→ ↑** EMT **→ ↓** cell adhesion **→ ↑** CSC Formation
Sanjari et al., 2020 [33]	↑ DNA Damage **→** ↑ p53 Mutation → **↑** WNT1 Formation **→ ↑** β-catenin **→ ↑** Cyclin D1 and c-Myc **→ ↑** CSC Self-Renewal

## Data Availability

Further inquiries can be directed to the corresponding authors.

## Abbreviations

The following abbreviations are used in this manuscript:

BPA	Bisphenol A, 2,2-Bis(4-hydroxyphenyl) propane
BCC	Breast Cancer Cell
CAT	Catalase (CAT) or Chloramphenicol acetyltransferase
COX-2	cyclooxygenase-2
CSC	Cancer Stem Cell
DCIS	Ductal Carcinoma In Situ
EDC	Endocrine Disrupting Compound
EMT	Epithelial–Mesenchymal Transition
ER	Estrogen Receptor
ERT	Estrogen Replacement Therapy
ETC	Electron Transport Chain
HER2	Human Epidermal Growth Factor Receptor 2
HIF-1α	Hypoxia-inducible factor 1-alpha
IDC	Invasive Ductal Carcinoma
ILC	Invasive Lobular Carcinoma
NF-κB	Nuclear factor kappa B
PGE2	Prostaglandin E2
PR	Progesterone Receptor
ROS	Reactive Oxygen Species
SOD	Superoxide dismutase
WNT1	WNT family member 1
ZEB1	Zinc finger E-box-binding homeobox 1
